# Supplementary material for: Flow-sensory contact electrification of graphene
Source: Nat Commun. 2021 Mar 19;12:1755. doi: 10.1038/s41467-021-21974-y (PMC7979811; doi:10.1038/s41467-021-21974-y)
Supplement: Supplementary file 1 — Supplementary Information [file 41467_2021_21974_MOESM1_ESM.pdf]

Flow-Sensory Contact Electrification of Graphene

Xiaoyu Zhang[1], Eric Chia[1], Xiao Fan[1], Jinglei Ping[1, 2, *]

Affiliations

[1]Department of Mechanical and Industrial Engineering, University of Massachusetts Amherst, Amherst, MA 01003, USA.

[2]Institute of Applied Life Sciences, University of Massachusetts Amherst, Amherst, MA 01003, USA.

These authors contributed equally: Eric Chia, Xiao Fan

*Corresponding author: Jinglei Ping

Email: ping@engin.umass.edu.

Table of Contents

Supplementary Table 1

Supplementary Figures 1-8

Supplementary References

Supplementary Table 1 | Summary of typical device-based flow sensors developed in previous researches.

Authors	Resolution/ Detection Limit (mm s^{-1})	Transduction Mechanism	Tested Fluid
Bertrand Bourlon, Marc Bockrath, *et al.*[1]	20.8	Streaming potential	NaCl solution
Rong Xiang He, Feng Yan, *et al.*[2]	4.62	Streaming potential	KCl solution
A. K. M. Newaz, K. I. Bolotin, *et al.*[3]	0.1	Streaming potential	NaCl solution
Dong Rip Kim, Xiaolin Zheng, *et al.*[4]	0.55-1.1	Streaming potential	KCl solution
Ying Chen, J. Iwan D. Alexander, *et al.*[5]	~3.0	Streaming potential	DI water
Shota Sando, Tianhong Cui, *et al.*[6]	0.59	Streaming potential	PBS
B. H. Son, Y. H. Ahn, *et al.*[7]	0.42	Streaming potential	DI water
Xiuhan Li, Zhong Lin Wang, *et al.*[8,*]	~0.2	Moving EDL boundary	Tap and DI water
Jun Yin, Wanlin Guo, *et al.*[9,*]	13	Moving EDL boundary	$NH_3 \cdot H_2O$, NaCl, $MgCl_2$, HCl solution, water
Jun Yin, Wanlin Guo, *et al.*[10,*]	22.5	Moving EDL boundary	LiCl, NaCl, KCl, HCl, NaF, NaBr
Trevor Hudson, Ellis Meng, *et al.*[11]	0.035	Calorimetry	PBS
Jaione Etxebarria, Aitor Ezkerra, *et al.*[12]	0.013	Calorimetry	DI water
Alex Baldwin, Ellis Meng, *et al.*[13]	0.019	Calorimetry	PBS
Ellis Meng, Yu-Chong Tai, *et al.*[14]	0.017	Calorimetry	DI water
R Ahrens, K Schlote-Holubek, *et al.*[15]	0.625	Calorimetry	Water, oil
Lingling Zhang, Xingzhong Zhao, *et al.*[16]	0.06	Piezoelectric effect	PBS

* Those flow-sensory approaches are not suitable for continuous flows.

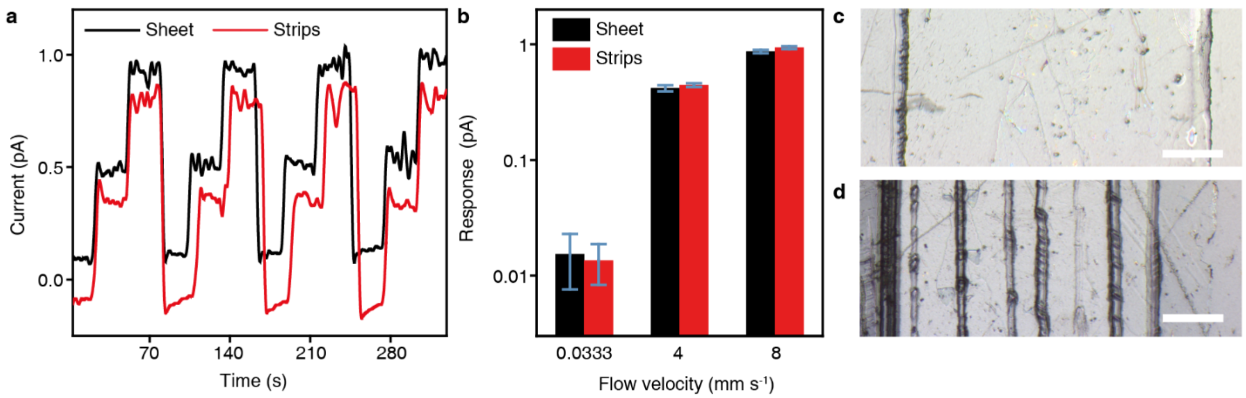

Supplementary Figure 1 | The charge transfer of graphene is minimally associated to the graphene edge electronic states. a The charge-transfer current that was measured by using a 1-mm wide sheet of graphene (with two edges) and that by using graphene stripes (with 16 edges) that were made from the graphene sheet by precision cutting. The syringe pump speed was changed in turn between 0.033, 4, and 8 mm s^{-1}. **b** The creation of the edges neither increases the calibration current magnitude nor the derivative of the current response with respect to flow velocity (the sensitivity). **c** The optical image of the graphene sheet prior to being cut. **d** The optical image of the graphene strips that were precision cut from the graphene sheet. The scale bars in **c** and **d** are 200 µm. The results suggest that the charge-transfer current and the current–flow association arise from the graphene basal plane electronic states.[17-19]

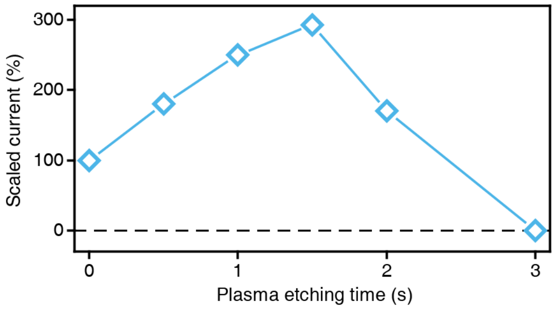

Supplementary Figure 2 | The charge-transfer current of graphene as a function of the period of oxygen-plasma treatment. The scale current is the charge-transfer current of a graphene microelectrode normalized by that of the graphene prior to oxygen plasma etching. The scale current was enhanced by up to 3× as the disorder density was increased by the period of plasma treatment, before the graphene was fully destroyed and the scaled current reached to zero. The sizes of the error bars (the standard deviations of scaled current based on the current-determination uncertainties over 1-Hz measurement bandwidth) are smaller than the size of the data points. The eye-guiding dash line is zero scaled current.

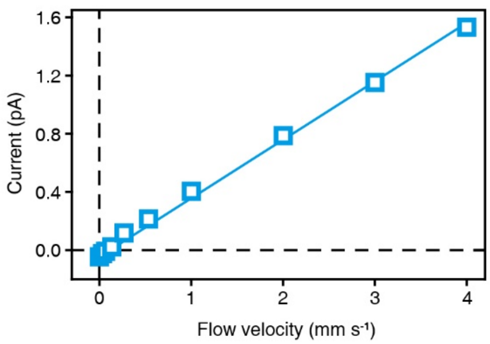

Supplementary Figure 3 | The charge-transfer current as a function of flow velocity. The current was obtained by proportional fit to the charge–time data (Fig. 1b in the main text). The solid line is the best linear fit to the data. The intersection of the eye-guiding dash lines is the origin of the coordinate system. The sizes of the error bars (the current standard deviations in 1-Hz bandwidth) are smaller than the size of the data points.

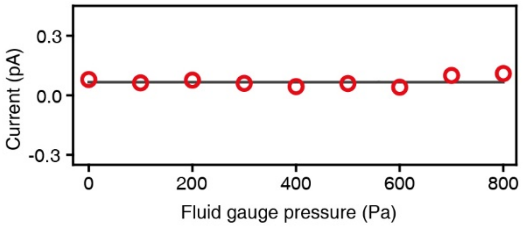

Supplementary Figure 4 | Charge-transfer current as a function of pressure difference for stagnant blood over a graphene device. The blood was kept motionless for each applied gauge pressure in the measurement. The black line is the best constant fit to the data. The fit value is 0.067 ± 0.008 pA. The sizes of the error bars (the current standard deviations in 1-Hz bandwidth) are smaller than the size of the data points. Since the maximum gauge pressure difference over the graphene device in our flow-varying charge transfer measurements is 665 ± 44 Pa (corresponding to a flow velocity of 8 mm s^{-1}), the flow-response current is minimally associated with potential piezoelectricity effects.[20]

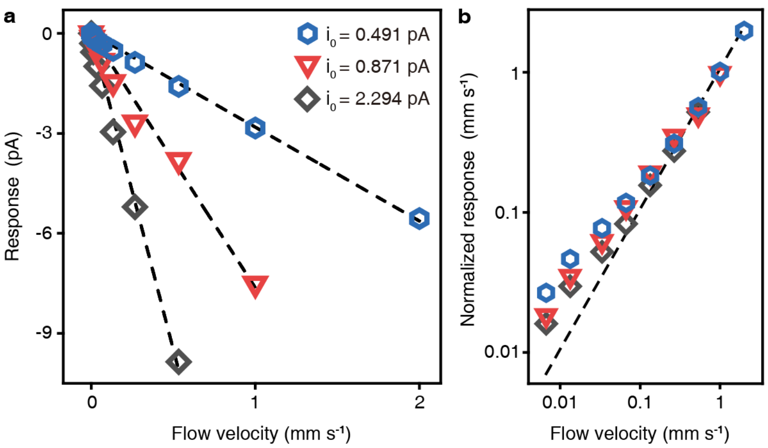

Supplementary Figure 5 | Current response of the graphene device in addition to Fig. 2a and b in the main text. a The current response as a function of flow velocity. The dash lines are best proportional fits to the data. **b** The sensitivity-normalized current response as a function of flow velocity. The eye-guiding dash line is of unit slope. The data symbols are the same as in **a**. In **a** and **b**, the sizes of the error bars (the standard deviations of corresponding quantities based on the current-determination uncertainties in 1-Hz bandwidth) are smaller than the size of the data points.

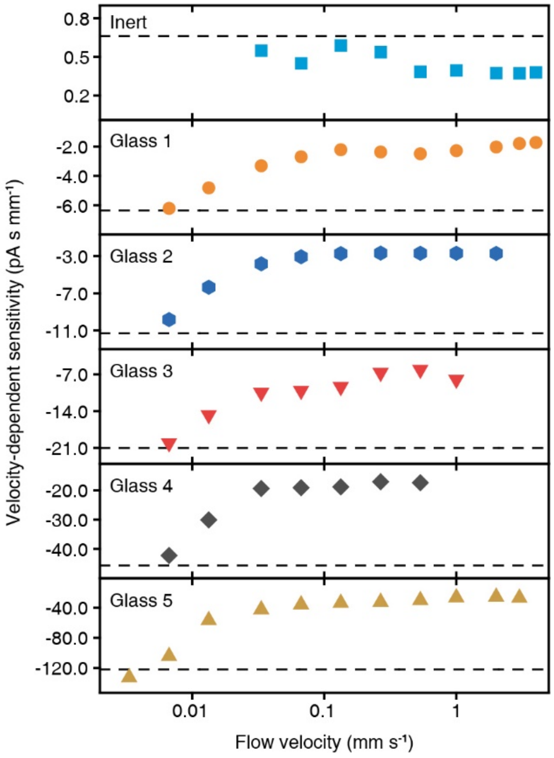

Supplementary Figure 6 | The velocity-dependent sensitivity for measurements with (Glass1–5) and without (Inert) using an unbiased glass electrode. The velocity-dependent sensitivity is obtained by taking numerical derivative of the current with respect to flow velocity at different flow velocities. The dash lines are the baseline velocity-dependent sensitivities (at zero flow velocity). The optimal resolution is obtained as the ratio of the standard deviation of the measured current to the absolute value of the baseline velocity-dependent sensitivity. The descriptions for the glass electrodes are the same as in Fig. 2**c** in the main text. The sizes of the error bars (the sensitivity standard deviations based on the current-determination uncertainties over 1-Hz bandwidth) are smaller than the size of the data points for all panels.

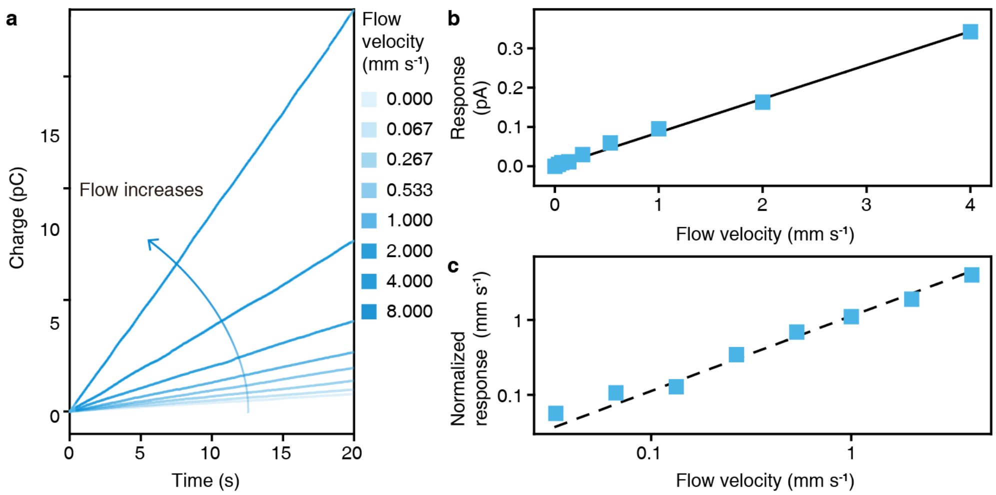

Supplementary Figure 7 | Real-time charge transfer, current response, and the normalized current between the graphene single microelectrode and PBS in measurement without using an unbiased glass electrode. **a** Real-time unsmoothed charge transfer. The graphene device is the same one that was used in the blood measurement. **b** The relationship between the current response and flow velocity. The black line is the best proportional fit to the data, which yields the fit parameter value of the slope (the sensitivity of the device) equal to 0.086 ± 0.002 pA s mm^{-1}, 0.22 ± 0.01 times of that for whole blood. The currents were extracted in a bandwidth of 1 Hz. **c** Log-log plot for the normalized current versus flow velocity. The eye-guiding dash line is of unit slope. The sizes of the error bars (the standard deviations of corresponding quantities based on the current-determination uncertainties in 1-Hz bandwidth) in **b** and **c** are smaller than the size of the data points.

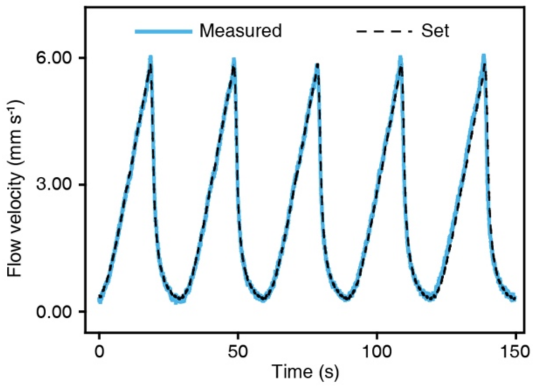

Supplementary Figure 8 | Monitored flow velocity in response to sawtooth-like PBS flow waveform driven by the syringe pump. The measured velocity responds with minimal delay to abrupt variations at the sharp apexes in the waveforms and the discrepancy of the flow velocity with the set velocity is minimal. The blue line represents the results of our real-time measurement using the graphene device. The dashed line represents the flow velocities set by the syringe pump.

Supplementary References

1. Bourlon, B., Wong, J., Mikó, C., Forró, L. & Bockrath, M. A nanoscale probe for fluidic and ionic transport. *Nature Nanotechnology* **2**, 104-107 (2007).
2. He, R. X. *et al.* Solution-gated graphene field effect transistors integrated in microfluidic systems and used for flow velocity detection. *Nano Letters* **12**, 1404-1409 (2012).
3. Newaz, A., Markov, D., Prasai, D. & Bolotin, K. Graphene transistor as a probe for streaming potential. *Nano Letters* **12**, 2931-2935 (2012).
4. Kim, D. R., Lee, C. H. & Zheng, X. Probing flow velocity with silicon nanowire sensors. *Nano Letters* **9**, 1984-1988 (2009).
5. Chen, Y., Liang, D., Gao, X. P. & Alexander, J. I. D. Sensing and energy harvesting of fluidic flow by InAs nanowires. *Nano Letters* **13**, 3953-3957 (2013).
6. Sando, S. & Cui, T. in *2017 19th International Conference on Solid-State Sensors, Actuators and Microsystems (TRANSDUCERS).* 1738-1741 (IEEE).
7. Son, B., Park, J.-Y., Lee, S. & Ahn, Y. Suspended single-walled carbon nanotube fluidic sensors. *Nanoscale* **7**, 15421-15426 (2015).
8. Li, X. *et al.* Self-powered triboelectric nanosensor for microfluidics and cavity-confined solution chemistry. *ACS Nano* **9**, 11056-11063 (2015).
9. Yin, J. *et al.* Generating electricity by moving a droplet of ionic liquid along graphene. *Nature Nanotechnology* **9**, 378-383 (2014).
10. Yin, J. *et al.* Waving potential in graphene. *Nature Communications* **5**, 1-6 (2014).
11. Hudson, T., Baldwin, A. & Meng, E. in *2019 IEEE 32nd International Conference on Micro Electro Mechanical Systems (MEMS).* 731-734 (IEEE).
12. Etxebarria, J. *et al.* Low cost polymeric on-chip flow sensor with nanoliter resolution. *Sensors and Actuators B: Chemical* **235**, 188-196 (2016).

13. Baldwin, A., Hudson, T. & Meng, E. in *2018 IEEE Micro Electro Mechanical Systems (MEMS).* 361-364 (IEEE).

14. Meng, E., Li, P.-Y. & Tai, Y.-C. A biocompatible Parylene thermal flow sensing array. *Sensors and Actuators A: Physical* **144**, 18-28 (2008).

15. Ahrens, R. & Schlote-Holubek, K. A micro flow sensor from a polymer for gases and liquids. *Journal of Micromechanics and Microengineering* **19**, 074006 (2009).

16. Zhang, L. *et al.* Highly sensitive microfluidic flow sensor based on aligned piezoelectric poly (vinylidene fluoride-trifluoroethylene) nanofibers. *Applied Physics Letters* **107**, 242901 (2015).

17. McCreery, R. L., Cline, K. K., McDermott, C. A. & McDermott, M. T. Control of reactivity at carbon electrode surfaces. *Colloids and Surfaces A: Physicochemical and Engineering Aspects* **93**, 211-219 (1994).

18. Yamada, Y., Miyazaki, K. & Abe, T. Role of edge orientation in kinetics of electrochemical intercalation of lithium-ion at graphite. *Langmuir* **26**, 14990-14994 (2010).

19. McCreery, R. L. Advanced carbon electrode materials for molecular electrochemistry. *Chemical Reviews* **108**, 2646-2687 (2008).

20. Ong, M. T. & Reed, E. J. Engineered piezoelectricity in graphene. *ACS Nano* **6**, 1387-1394 (2012).
