# Supplementary material for: Flow-sensory contact electrification of graphene
Source: Nat Commun. 2021 Mar 19;12:1755. doi: 10.1038/s41467-021-21974-y (PMC7979811; doi:10.1038/s41467-021-21974-y)
Supplement: Supplementary file 3 — Description of Additional Supplementary Files [file 41467_2021_21974_MOESM3_ESM.pdf]

Description of Additional Supplementary Files

Title: Supplementary Movie 1

Description: The program for real-time signal acquisition and data processing. The panel at the top right corner shows the electrical current that is numerically extracted from the transferred charge (lower right corner) of a graphene singlemicroelectrode device in real time. The current is smoothed by a realtime digital S-G filter (in a 1-Hz bandwidth). The left panel shows the flow velocity that is translated from the smoothed current by implementing interpolation of the corresponding current–flow data set.
